# Protection against Different Genotypes of Newcastle Disease Viruses (NDV) Afforded by an Adenovirus-Vectored Fusion Protein and Live NDV Vaccines in Chickens

**DOI:** 10.3390/vaccines9020182

**Published:** 2021-02-21

**Authors:** Helena L. Ferreira, Patti J. Miller, David L. Suarez

**Affiliations:** 1 Southeast Poultry Research Laboratory, US National Poultry Research Center, USDA-ARS, 934 College Station Rd., Athens, GA 30605, USA; hlage@usp.br (H.L.F.); pjmdvm97@uga.edu (P.J.M.); 2 Department of Veterinary Medicine, FZEA-USP, University of Sao Paulo, Pirassununga, SP 13635-900, Brazil; 3 Department of Population Health, College of Veterinary Medicine, University of Georgia, Athens, GA 30602, USA

**Keywords:** vaccines, NDV, poultry, fusion, homologous, heterologous, immunity

## Abstract

The efficacy of an adenovirus-vectored Newcastle disease virus (NDV) vaccine expressing the fusion (F) NDV protein (adeno-F) was evaluated against challenges with virulent heterologous and homologous NDV strains to the F protein. In a preliminary study, two different doses (low and high) of adeno-F were tested against a virulent NDV strain containing the homologous NDV F protein, CA02. In a second study, at three weeks post-vaccination, the efficacy of the high dose of adeno-F was compared to a live attenuated NDV vaccine strain (LaSota) against three antigenically distinct virulent NDV challenge strains, one homologous (CA02) and two heterologous (TZ12, EG14) to F in the vectored vaccine. In both experiments, clinical signs, mortality, virus shedding, and humoral response were evaluated. In the first experiment, the survival rates from birds vaccinated with adeno-F at a high and low dose were 100% and 25%, respectively. In the second experiment, birds vaccinated with the high dose of adeno-F had a survival rate of 80%, 75%, and 65% after challenge with the CA02, TZ12, and EG14 viruses, respectively. All of the LaSota-vaccinated birds survived post-challenge no matter the NDV challenge strain. High antibody titers were detected after vaccination with LaSota by HI and ELISA tests. The majority of adeno-F-vaccinated birds had detectable antibodies using the ELISA test, but not using the HI test, before the challenge. The data show that both the similarity of the F protein of the adeno-F vaccine to the challenge virus and the adeno-F vaccination dose affect the efficacy of an adenovirus-vectored NDV vaccine against a virulent NDV challenge.

## 1. Introduction

Newcastle disease virus (NDV) is distributed worldwide, and virulent strains often cause severe disease in poultry [1]. Member countries that identify virulent NDV isolates in poultry must immediately report to the World Organisation for Animal Health (OIE), and trade restrictions on poultry or poultry products may be imposed by other countries [2]. NDV (used hereafter and also known as avian paramyxovirus 1, APMV-1) belongs to the family *Paramyxoviridae*, subfamily *Avulavirinae*, genus *Orthoavulavirus*, species *avian orthoavulavirus 1* [3]. The virus has a lipid bilayer envelope with a single-stranded, negative-sense, non-segmented RNA genome organized as follows: 3′—nucleoprotein (NP)—phosphoprotein (P)—matrix protein (M)—fusion protein (F)—hemagglutinin-neuraminidase (HN)—large polymerase protein (L)—5′. Each viral gene encodes for a single structural protein with the same name and two additional nonstructural proteins, named V and W, are produced by RNA editing of the P gene [4]. The lipid bilayer envelope is derived from the plasma membrane of the host cell possessing two integral glycoproteins: fusion (F) and hemagglutinin-neuraminidase (HN) [5].

The HN glycoprotein is involved in cell attachment and has neuraminidase (NA) enzymatic activities. The HN protein can elicit neutralizing antibodies and it is the protein that has hemagglutinating activity [5]. The stalk region of HN also affects the fusion due to its interaction with the F protein [6]. The F protein is critical for release of viral RNA into the cell and mediates a pH-independent fusion of the viral envelope with the plasma membrane of the host cell. In late infection, infected cells expressing F proteins are present at the plasma membrane. These proteins can mediate fusion with neighboring cells to form syncytia (giant cell formation), a cytopathic effect that can lead to tissue necrosis in vivo and might also be a mechanism of virus spread [5]. Interestingly, a large syncytium requires the expression of homologous HN and F proteins [7]. Studies showed that the stalk, but not the globular head, of the HN protein triggers the F protein and can promote cell fusion [8,9]. Some amino acids in the F protein can increase the tropism to the brain, spleen, and lung, but the additional inclusion of the HN protein substantially increases the tropism [7]. These two outer surface glycoproteins of enveloped viruses [10,11,12] and other proteins [13] have also been reported to enhance viral virulence.

All NDV isolates are classified into a single serotype; however, they are genetically and antigenically diverse. NDV strains that primarily cause outbreaks in poultry belong to class II and they are divided into at least 20 genotypes (named I to XXI, but excluding XV) with genetic distances greater than 10% between genotypes, and some of them are further divided into sub-genotypes [14]. This genetic variation resulting from point mutations when in antigenic sites on the glycoproteins (HN and/or F) may affect the protection induced by live, inactivated, or recombinant vaccines against the circulating NDV strain.

The clinical protection induced by the HN or F glycoproteins against NDV has been evaluated by using different vaccine platforms, such as viral vector (adenovirus, fowlpox, herpesvirus of turkeys (HVT), baculovirus) and DNA vaccines [15,16,17,18,19,20]. Regardless of the platform, an increase in protection is usually detected when both HN and F are used together when compared to the protection induced by vaccines that utilize F or HN alone [2,21]. Interestingly, the protection induced by F is usually better than HN alone [15,17,22], despite the great importance of HN for virus attachment, neuraminidase, and fusion activities [23]. Clinical protection can also be improved by using an NDV vaccine antigenically matching the challenge virus [24,25,26]. The efficacy of using vaccines homologous or heterologous to challenge strains has been evaluated using recombinant and/or inactivated vaccines by our group [24,25]. For example, inactivated or recombinant vaccines with modified fusion cleavage sites (to decrease the virulence) homologous to the NDV challenge induced stronger humoral systemic responses and resulted in smaller amounts of challenge virus shed after challenge compared to vaccines formulated with heterologous NDV strains [26,27].

The first objective of these studies was to evaluate how the concentration of the F protein (dose) of an adenovirus-vectored NDV vaccine expressing the F protein of NDV (adeno-F) affects the protection against a homologous virulent NDV challenge to the F protein of the vaccine. The second objective was to evaluate the protection induced by the adeno-F vaccine against various heterologous challenges as would be expected in a field setting while also using a traditional live NDV vaccine strain as a control.

## 2. Materials and Methods

### 2.1. Chickens

In total, 143 three-week-old specific pathogen-free (SPF) White Leghorn (WL) chickens (*Gallus gallus domesticus*) were obtained from the Southeast Poultry Research Laboratory (SEPRL) flock. Feed and water were provided with ad libitum access. Birds were kept in isolators, and the animal experiments were approved and performed in accordance with the Institutional Animal Care and Use Committee (IACUC) in animal biosecurity level 3 enhanced (ABSL-3E) facilities at the SEPRL.

### 2.2. Viruses

Three NDV viruses obtained from the SEPRL virus repository were used to perform the virus challenge: NDV gamefowl/California/212676/2002 (CA02), chicken/Egypt/Sohag/18/1020/2014 (EG14), chicken/Tanzania/Tanga/N38/1317/2012 (TZ12) [28,29,30,31]. Viruses were propagated in SPF embryonating chicken eggs (ECE), as previously described [32]. Virus-infected allantoic fluid was diluted in brain heart infusion (BHI) medium (BD Bioscience, Sparks, MD) to obtain an inoculum with titers of 5.7 and 6.5 log_10_ 50% egg infectious dose (EID_50_) per bird. The LaSota/1946 virus strain was used for vaccination with titers of 7 log_10_ EID_50_/mL per bird and also used for the immunofluorescent assays.

### 2.3. Adenovirus Construct

Human adenovirus serotype 5, a replication-restricted vector system, particles containing the F gene from the NDV gamefowl/California/212676/2002 sequence with a genetic modification from polybasic to monobasic in the F cleavage site under a CMV promoter were commercially produced (VectorBuilder, Cyagen Biosciences Inc, Santa Clara, USA). The construct was used for bird vaccination at 8 (low dose) and 9 log_10_ (high dose) PFU/mL concentrations by the intra-muscular route diluted in phosphate-buffered saline (PBS).

### 2.4. Immunofluorescent Assay (IFA)

The immunofluorescent assay was performed to confirm the adeno-F expression. In a 12-well plate, 5.5 log_10_ cells/mL of MDCK cells were plated in DMEM supplemented with 10% fetal bovine serum (FBS), penicillin (10,000 units/mL), streptomycin (10,000 µg/mL), and amphotericin B (25 µg/mL) (Gibco, Carlsbad, USA). After 24 h, the medium was removed, and cells were infected with NDV LaSota using multiplicity of infection (MOI) at 0.1 or transduced with adenovirus expressing the F gene using MOI of 10. After 90 min to allow adsorption on the MDCK cells, new media were added with 2 % fetal bovine serum (FBS) and 1× antibiotics (Invitrogen, Carlsbad, CA, USA). Cells infected with LaSota virus were also supplemented with 10% allantoic fluid from SPF ECE to allow the virus replication. After 48 h, media were removed, and cells were fixed using 10% formalin for 15 min. Monolayer cells were washed with PBS and 100% cold methanol was used for 10 min at 20 °C to permeabilize cells. Blocking was conducted using 5% bovine serum albumin (BSA) in PBS supplemented with 0.1% Tween 20 (PBST) for 1 h at room temperature (RT). After that, primary rabbit anti-F protein antibody (#5057, kindly provided by Dr. Claudio Afonso, SEPRL) was added at 1:100 in diluted PBST for 1 h at RT. After the wash step using PBS, goat anti-rabbit IgG, FITC conjugated antibody (SouthernBiotech, Birmingham, AL, USA) was diluted at 1:200 in PBS with Tween 20 (PBST) for 1 h at RT. After the wash step, monolayers were visualized under the EVOS FL fluorescent microscope (Thermo Fisher Scientific, Carlsbad, CA, USA).

### 2.5. Phylogenetic Analysis

Total RNAs were extracted using Trizol LS (Ambion-Thermo Fisher Scientific, Carlsbad, USA) from the three isolates (CA02, EG14, TZ12) and were quantified by Qubit fluorimetry (Thermo Fisher Scientific, Carlsbad, USA). RNA was reverse-transcribed, and DNA libraries for next-generation sequencing were prepared, sequenced, and analyzed as described previously [33]. Raw sequence data were analyzed and assembled using MIRA version 3.4.1 within a customized workflow on the Galaxy platform as described previously [34]. The CA02, EG14, and TZ12 sequences are available in GenBank under the accession numbers EF520718, MH392219, and MK673140, respectively.

Seventy-six complete fusion gene-coding sequences related to the viruses studied here and representative sequences from other genotypes from class II NDV genotypes were downloaded from GenBank. The model for phylogenetic analysis was selected based on the lowest Bayesian information criterion scores (BIC) and Akaike information criterion corrected value (AICc), which are considered to describe the substitution pattern the best. The coding regions of the complete fusion gene were used to construct final phylogenetic trees using MEGA7 [35]. The general time reversible (GTR) with a discrete gamma distribution (5 categories [+G, parameter = 1.8054]) and assuming that a certain fraction of sites is evolutionarily invariable (+I) was utilized for the complete fusion gene tree. Pairwise distances based on deduced amino acid sequences were also estimated. The current NDV classification criteria for genotype and sub-genotype identification were followed in this study [14].

### 2.6. Experimental Design

Two experiments were performed (Figure S1). In the first experiment, 21 chickens were split into 3 groups: (1) control (*n* = 5); (2) adeno-F at a high dose (10^9^ PFU/mL, *n* = 8); and (3) adeno-F at a low dose (10^8^ PFU/mL, *n* = 8). Three weeks after vaccination, the birds were challenged with CA02 through the oculo-nasal route at 10^6.5^ EID_50_ in 100 µL per bird using gavage needles, and the inoculum was delivered via the choanal cleft and the left eye of each bird. In the second experiment, 120 birds were divided into 9 groups. Initially, 30 birds were vaccinated with the LaSota strain and 60 birds were vaccinated with adeno-F at a high dose, and 30 non-vaccinated birds were used as a control. Three weeks after vaccination, ten birds from the control and LaSota groups and birds vaccinated with adeno-F were challenged using three different NDV isolates (CA02, EG14, and TZ12) at 10^5.7^ EID_50_ in 100µl per bird through the oculo-nasal route (20 birds per isolate). In both experiments, clinical signs and mortality were monitored for 11 to 14 days after challenge. Oropharyngeal (OP) and cloacal (CL) swabs were collected from all birds at 2, 4, and 7 days post-challenge (dpc) to determine virus shedding. Blood was collected before and after each challenge (end of the experiment) to evaluate the humoral response. The mean death time (MDT) was calculated by averaging the day individual birds died from challenge in each group or were euthanized and reported as dead on the next day.

### 2.7. Virus Shedding

OP and CL swabs were collected in 2 mL of BHI broth with a final concentration of 10 μg/mL of gentamicin, 100 units/mL of penicillin G, and 56 μg/mL of amphotericin B and kept frozen at −70 °C until processing. RNA was extracted using the MagMax AI/ND RNA isolation kit (Ambion, Inc., Austin, USA) [36]. Quantitative real-time RT-PCR (RRT-PCR) targeting the M gene (M-4100 test) for NDV detection was performed as previously described [37]. In the second experiment, RRT-PCR targeting the polymerase gene (L-12200 test) was used as previously described [28]. All RRT-PCR reactions were carried out on an ABI 7500 (Applied Biosystems Carlsbad, USA). A standard curve for virus quantification was established with 10-fold serial dilutions in nuclease-free water of RNA extracted from titrated challenge viruses, and results were reported as EID_50_/mL equivalents. The calculated RRT-PCR lower detection limit for the viruses using the M-4100 test was 2.5 log_10_ EID_50_/mL and it varied between 1.5 and 1.7 log_10_ EID_50_/mL using the L-12200 test.

### 2.8. Hemagglutination Inhibition (HI) Test

The HI test was used to quantify antibody responses after vaccination and challenge as previously described [38]. Serum was collected from all birds in both experiments at 18 or 21 days post-vaccination (dpv, pre-challenge) and from surviving birds at the end of the experiment using the homologous antigen to the challenge. All collected sera were tested by the HI test, except the pre-challenge in experiment 2 when only groups vaccinated with the LaSota vaccine were tested. Titers were calculated as the highest reciprocal serum dilution providing complete hemagglutination inhibition. Serum titers equal or above 4 Log2 were considered as positive.

### 2.9. Indirect ELISA for Detection of Antibodies Specific to NDV

The ID screen Newcastle disease indirect ELISA test (IDvet, Grables, France) was used to assess the antibody titers against NDV before the challenge in the second experiment. Two different dilutions 1/100 and 1/500 (the latter recommended by the manufacturer) of the sera collected before the challenge and only one dilution 1/500 of the sera from after challenge were tested. The test and the titer calculation were performed according to the manufacturer’s recommendations. Titers above 993 were considered as positive.

### 2.10. Statistical Analysis

Data were analyzed using Prism (v.7.03) software and outliers were identified using the ROUT test (GraphPad Software Inc., La Jolla, USA). Survival curves were tested using the log-rank (Mantel–Cox) test with a significance level of *p* < 0.0001. For virus shedding, the D’Agostino–Pearson normality test was performed to estimate if the values in each group come from a Gaussian distribution. Based on the normality distribution, the parametric ANOVA test was used for multiple comparisons of mortality rates and viral titers in OP or CL swab samples from the different treatments (control, adeno-F—low and high doses, and LaSota) on the same day and with the same NDV challenge strain. Statistical significance was set at a *p*-value of ˂0.05. The means ± standard errors with no common letters differ significantly. HI titers were tested using one-way ANOVA. HI or ELISA antibody titers in sera samples from the different treatments (control, adeno-F, and LaSota) and NDV challenge strains were compared. Pearson correlation was performed to evaluate the correlation between the virus titers measured by the ELISA test and virus shedding measured at 4 dpc by the oral route.

## 3. Results

### 3.1. IFA

Positive staining was observed in MDCK cells transduced with adeno-F (Figure S2). The positive control using cells infected with LaSota NDV was also detected, while the non-infected cells did not show any fluorescence.

### 3.2. Phylogenetic Analysis

**Figure 1:**
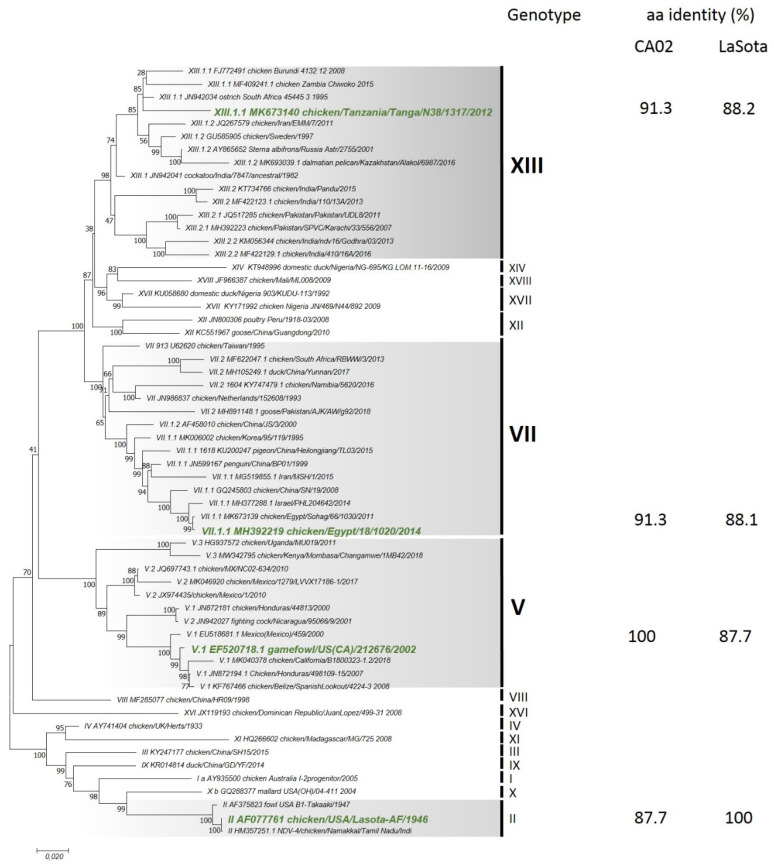
Phylogenetic analysis of challenge strains and amino acid identity of vaccines compared to challenge strains (names highlighted in green and italicized). The evolutionary history was inferred by using the maximum likelihood method based on the general time reversible model [21]. The tree with the highest log likelihood (−18,610.06) is shown. The percentage of trees in which the associated taxa clustered together is shown next to the branches. Initial tree(s) for the heuristic search was (were) obtained automatically by applying neighbor joining and BioNJ algorithms to a matrix of pairwise distances estimated using the maximum composite likelihood (MCL) approach and then selecting the topology with a superior log likelihood value. A discrete gamma distribution was used to model evolutionary rate differences among sites (5 categories (+G, parameter = 0.4368)). The tree is drawn to scale, with branch lengths measured in the number of substitutions per site. The analysis involved 59 nucleotide sequences. All positions containing gaps and missing data were eliminated. There were a total of 1656 positions in the final dataset. Evolutionary analyses were conducted in MEGA7 [35].

The phylogenetic analysis performed with the coding sequence of the fusion gene showed that the three challenge viruses cluster into three different class II genotypes (Figure 1). The CA02 virus clustered with viruses isolated in Central and North America belonging to sub-genotype V.1 isolated from 2002 to 2008. The EG14 clustered with other viruses from Egypt, Israel, and China, and it belongs to sub-genotype VII.1.1. The TZ12 virus clustered with isolates from different African countries that were isolated from 2008 to 2015, and it belongs to sub-genotype XIII.1.1. The deduced amino acid identities of the CA02 fusion gene in adeno-F and the EG14 and TZ12 fusion amino acid sequences were 91.3%. The homology of the fusion amino acid sequence from the LaSota virus and the three challenge viruses varied from 87.7 to 88.2%.

The sequences of all three challenge viruses had three basic amino acids between residues 113 and 116 in the C-terminus of the F2 protein and a phenylalanine at residue 117 in the *n*-terminus of the F1 protein (^113^RQKR↓F^117^).

### 3.3. Clinical Protection

**Figure 2:**
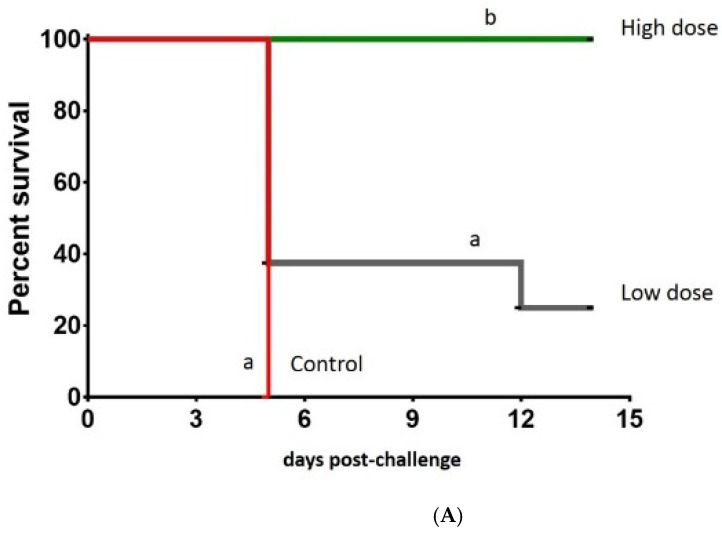

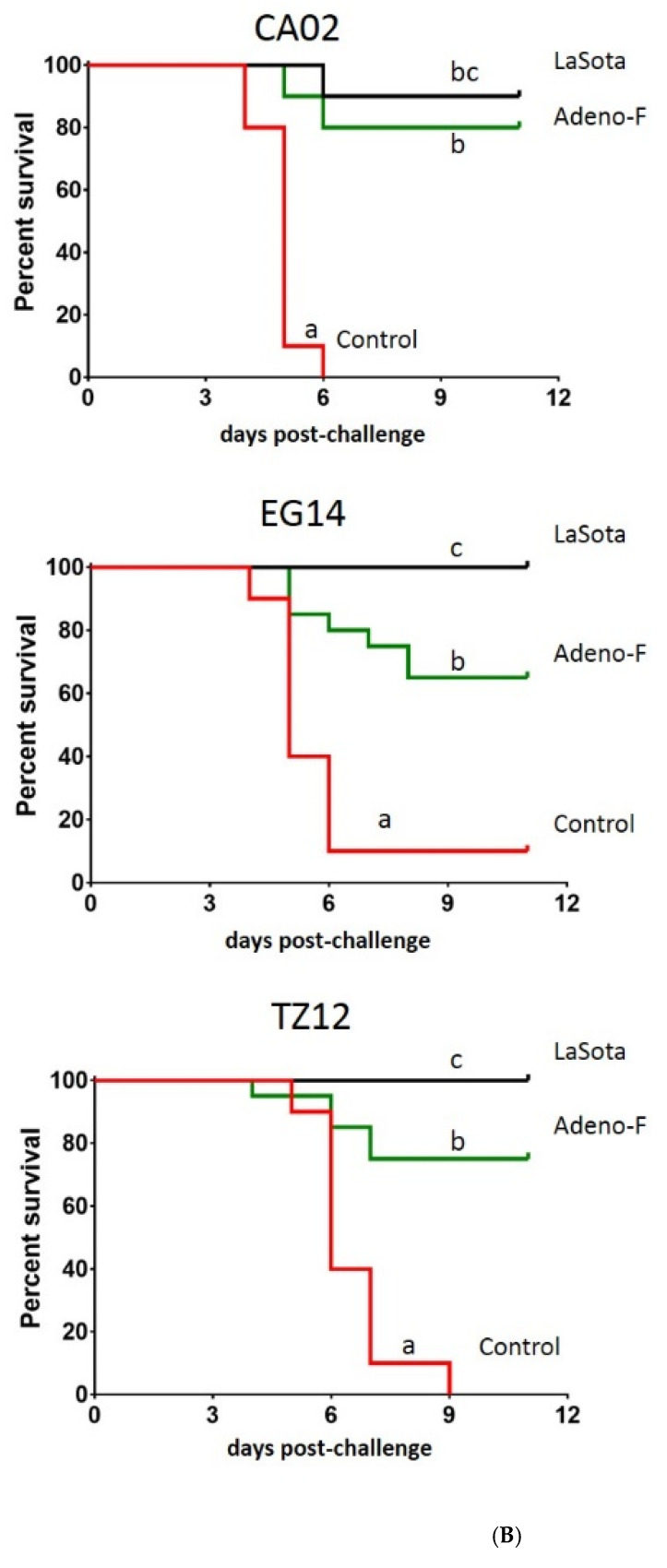
Survival rates after Newcastle disease virus (NDV) challenge in control and vaccinated birds. (**A**) Vaccinated birds with the low and high doses of adeno-F and control groups were monitored after NDV challenge with CA02 virus for 14 days. (**B**) Birds vaccinated with adeno-F and LaSota virus and non-vaccinated birds (control) after NDV challenge with CA02, EG14, and TZ12 viruses for 11 days. Different letters are statistically significant (*p* < 0.05).

In the first experiment, clinical signs typical of Newcastle disease were observed in birds from the control and adeno-F low dose, but not in birds vaccinated with adeno-F at a high dose. The survival rates from birds vaccinated with adeno-F at a high and low dose were 100% and 25%, respectively (Figure 2A). The MDT in the control and low-dose adeno-F groups was 5 and 6 dpc, respectively.

In the second experiment, all birds in the control groups displayed typical clinical signs of Newcastle disease, such as moderate to severe lethargy, ruffled feathers, diarrhea, and conjunctivitis at 3–4 dpc. All birds, with one exception, in the control groups succumbed to the virulent NDV challenge (Figure 2B). The MDT in control groups challenged with CA02, EG14, and TZ12 viruses was 4.9, 5.2, and 6.5 dpc, respectively.

As for birds vaccinated with LaSota, no clinical signs or mortality were observed, with the exception of one bird that died due to unrelated problems to the challenge. In the second experiment, 40% of all birds vaccinated with adeno-F and challenged displayed lethargy, ruffled feathers, diarrhea, and conjunctivitis at 5–6 dpc, labored breathing, and mucoid nasal discharge at 5–7 dpc. Additionally, head tremors and ataxia were observed in one bird each in the CA02 and EG14 groups and two birds challenged with the TZ12 virus at 8 to 11 dpc. The lowest morbidity rate (30%) and the highest survival rate (80%) were found in birds that received adeno-F and were challenged with the homologous CA02 virus (Figure 2B). The morbidity found in adeno-F-vaccinated birds challenged with the heterologous strains was slightly higher (40% to 50% for TZ12 and EG14 viruses, respectively) compared to the CA02 virus. The survival rates in the adeno-F groups after challenge with EG14 and TZ12 viruses were 65% and 75%, respectively. The MDT in the groups vaccinated with adeno-F and challenged with CA02, EG14, and TZ12 viruses were 4.6, 5.2, and 6.5 dpc, respectively.

No significant difference in survival was observed when comparing birds vaccinated with LaSota to those vaccinated with adeno-F when challenged with CA02, but they were different from the control birds (*p* < 0.05). However, birds vaccinated with the LaSota vaccine had significant differences in survival rates compared to control birds and birds vaccinated with adeno-F after challenge with EG14 and TZ12 viruses.

### 3.4. Virus Shedding

**Figure 3:**
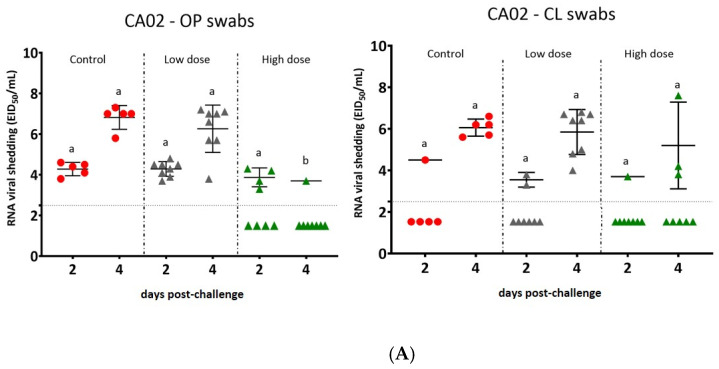

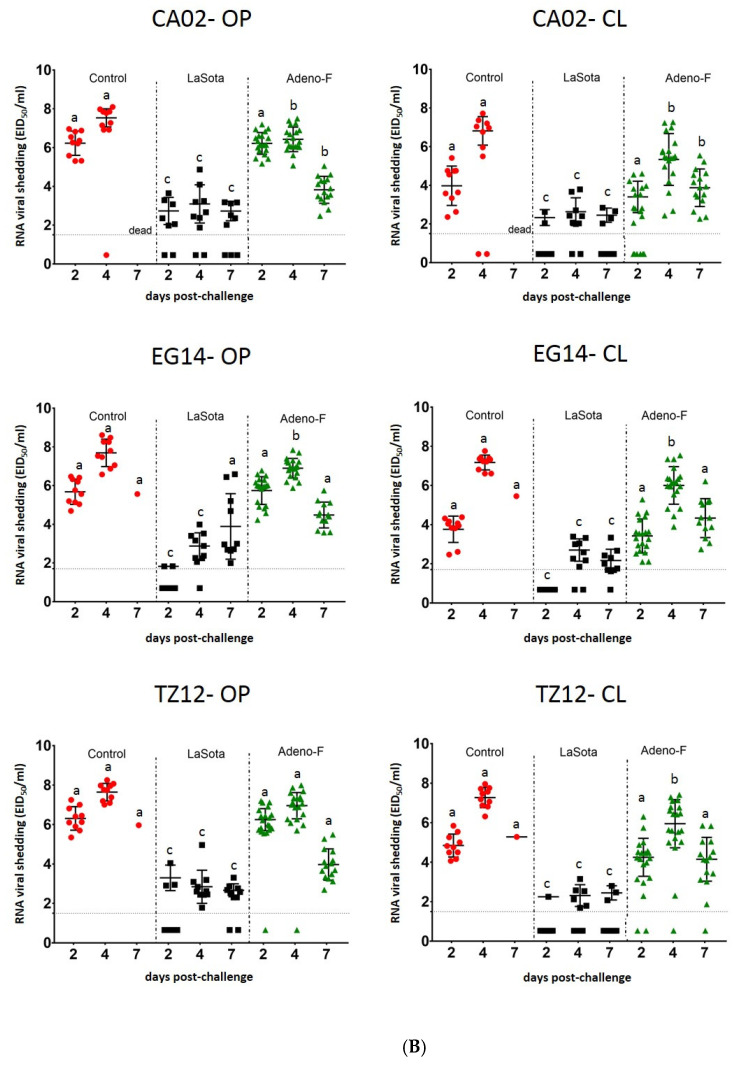
Vaccinated and non-vaccinated birds shed virus after NDV challenge in the different experiments. (**A**) Oropharyngeal (OP) and cloacal (CL) swabs from birds in the control group and in groups vaccinated with adeno-F at high and low doses were tested by RRT-PCR after 2 and 4 days post-challenge (dpc). (**B**) Virus shedding from vaccinated birds with LaSota and adeno-F vaccines and non-vaccinated birds in OP and cloacal swabs at 2, 4, and 7 days after challenge with CA02, EG14, or TZ12. Viral titers in OP or CL swab samples from the different treatments (control, adeno-F—low and high doses, and LaSota) on the same day and with the same NDV challenge strain. The mean ± standard errors with different letters (a, b, or c) are statistically significant (*p* < 0.05). CL: cloacal swab; OP: oropharyngeal swab.

In the first experiment, control birds and birds vaccinated with the low-dose adeno-F shed similar virus titers at 2 and 4 dpc by OP and CL swabs after the challenge with CA02 virus (Figure 3A). The peak of virus shedding in control birds and birds vaccinated with adeno-F at a low dose was measured at 4 dpc by OP swabs with virus titers of 6.8 and 6.3 log_10_ EID_50_/mL, respectively. Birds vaccinated with adeno-F at a high dose shed significantly lower (*p* < 0.05 = 0.0002) amounts of virus than controls birds at 4 dpc by OP swabs. Birds vaccinated with this vaccine at the high dose and challenged with CA02 virus had an average decrease of 2.3 log_10_ EID_50_/mL in the virus titer from the oropharynx when compared with controls at 4 dpc. At this time point, no significant difference was detected when comparing titers from cloacal swabs among all groups. However, all birds from the control and low-dose groups, but only three birds in the high-dose group, had detectable virus.

In the experiment using three different challenge NDV genotypes, all control birds shed significantly higher titers than all groups in most of the time points with a few exceptions (Figure 3B). In control birds, the peak of virus shedding was at 4 dpc with virus titers at 7.5, 7.6, and 7.6 log_10_ EID_50_/mL by OP swabs after the challenge with CA02, EG14, and TZ12 viruses, respectively. No significant difference in virus shedding was observed when comparing the virus shedding from control birds after the challenge with the three NDV isolates.

The virus titers in birds vaccinated with LaSota and challenged with each of the three virulent NDV strains were at least 3.8 log_10_ EID_50_/mL lower than virus titers shed by control birds. The peak of virus shedding was also mainly at 4 dpc from the OP and CL swabs after the challenge with the three viruses with some minor variations. For example, birds challenged with EG14 shed the highest virus titer (3.9 log_10_ EID_50_/mL) from the oropharynx at 7 dpc.

The birds vaccinated with adeno-F and challenged with the homologous strain, CA02, shed significantly less virus, 1.1 and 1.5 log_10_ EID_50_/mL, than controls at 4 DPC by oral and cloacal routes, respectively (*p* < 0.05 = 0.01 and 0.008). Birds challenged with CA02 shed lower virus titers than the other birds challenged with EG14 and TZ12 (*p* < 0.05 = 0.049). Birds challenged with EG14 virus also shed less virus, 0.8 and 1.2 log_10_ EID_50_/mL, than controls at 4 dpc by oral and cloacal routes, respectively. Birds challenged with TZ12 shed significantly less virus (1.3 log_10_ EID_50_/mL) than controls only at 4 dpc in cloacal swabs.

Birds vaccinated with the LaSota vaccine shed significantly lower amounts of virus (*p* < 0.05) than the birds vaccinated with adeno-F and the control birds at all time points by both OP and CL routes, except after the challenge with EG14 virus at 7 dpc by the oral route (*p* > 0.05), when the birds vaccinated with the LaSota vaccine shed the highest virus titer.

### 3.5. Serology

**Figure 4:**
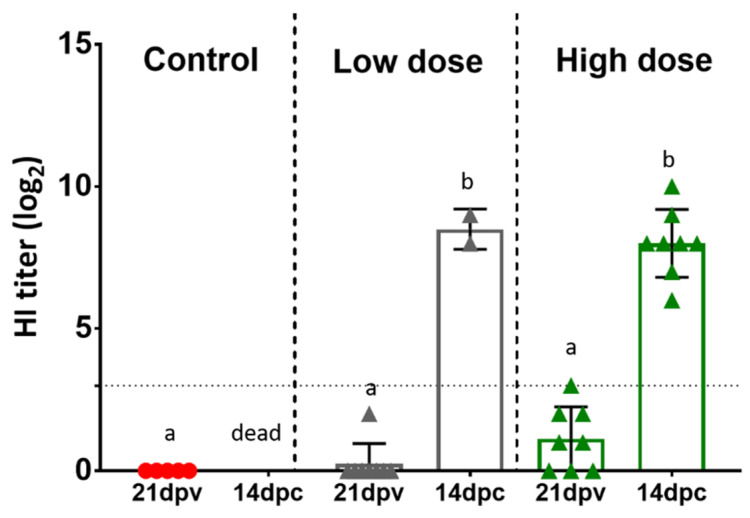
Antibody titers before and after challenge with CA02 virus. The hemagglutination inhibition (HI) test was performed in sera from non-vaccinated birds and birds vaccinated with adeno-F at low and high doses before and after challenge with CA02. No significant difference was detected comparing all groups before or after challenge. Different letters (a or b) are statistically significant. dpv: days post-vaccination, dpc: days post-challenge.

No HI antibody titer was detected in sera from birds vaccinated with adeno-F at the low and high doses at 3 weeks post-vaccination. All surviving birds had high HI titers specific to NDV at 14 days post-challenge (Figure 4).

As expected, no specific antibody to NDV was detected in any bird in control groups by the HI or ELISA test before the challenge. After 11 dpc, the remaining bird in the control group challenged with EG14 had an HI titer of 11 log_2_.

**Figure 5:**
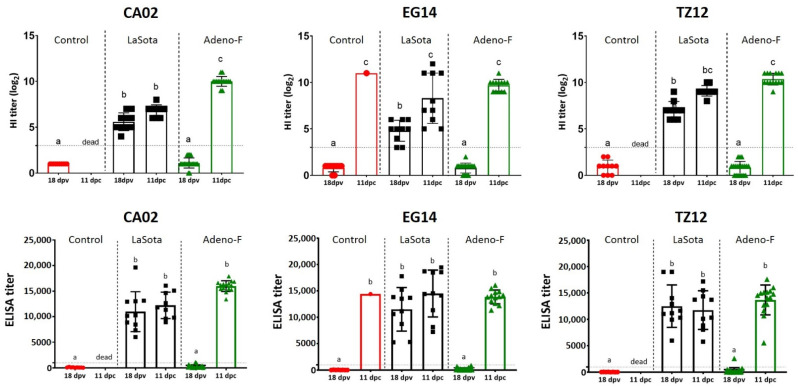
Antibody titers before and after challenge with NDV challenge strains. HI and ELISA tests were performed before and after the challenge using the homologous strains (CA02, EG14, and TZ12) in sera from control birds and birds vaccinated with LaSota or adeno-F vaccines. HI or ELISA antibody titers in sera samples from the different treatments (control, adeno-F, and LaSota) and NDV challenge strains were compared. Different letters (a, b or c) are statistically significant (*p* < 0.05). dpv: days post-vaccination, dpc: days post-challenge.

All birds vaccinated with LaSota virus, except two birds in the EG14 challenge group, had antibodies against NDV before the challenge using the homologous antigen to the challenge by the HI test (Figure 5). The birds vaccinated with LaSota and challenged with CA02 and EG14 viruses had HI antibody titers significantly lower (5.6 and 4.8 log_2_, respectively) than birds in the TZ12 challenge group (7 log_2_). The differences in HI titers were likely related to the different challenge antigens used for testing. At 11 dpc, the HI antibody titers slightly increased in all remaining birds in the LaSota groups, but the lowest HI titers were detected in birds challenged with CA02 virus (6.8 log_2_), which were significantly lower than sera from birds challenged with EG14 virus (8.3 log_2_).

All birds vaccinated with LaSota virus had high titers detected by the ELISA test before and after the challenge (Figure 5). The mean of titers ranged from 19,925 to 20,589 at 1/100 dilution and 12,903 to 14,774 at 1/500 dilution before the challenge. At 11 dpc, the ELISA titers slightly increased in surviving birds in these groups.

The surviving birds vaccinated with adeno-F had high HI antibody titers varying from 9.7 to 10.4 log_2_ when using the homologous antigen to the challenge by the HI test. Less than half (41.7%) of the vaccinated birds with adeno-F had ELISA titers above the positive threshold when tested using the 1/100 dilution before the challenge with the different viruses (Table 1). Ten, nine, and six birds from groups challenged with CA02, EG14, and TZ12 viruses, respectively, had antibodies against NDV at the 1/100 dilution. When tested using the recommended dilution by the manufacturer (1/500), only two birds in the CA02 group and one bird in the TZ12 group had positive antibodies. After the challenge, all the birds had high positive ELISA titers (Figure 5).

### 3.6. Correlation of Clinical Signs, Mortality, Virus Shedding, and Serology

All birds in the control groups did not have positive antibodies and succumbed to the NDV infection, with the exception of one bird in the EG14 group. Conversely, the birds vaccinated with the LaSota vaccine had high titers by ELISA and HI tests, and no mortality or clinical signs were recorded. These birds shed a significantly less amount of virus than the controls. Table 1 summarizes the individual data from mortality, clinical signs, and antibody response using the ELISA test with two different sera concentrations in the groups vaccinated with adeno-F.

As for the birds vaccinated with adeno-F and challenged with CA02 virus, the two birds with positive antibodies at 1/500 dilutions at 18 dpv did not show any clinical sign. Ten birds in the same group had detectable antibodies at 1/100 dilution at 18 dpv by ELISA, but two of them displayed clinical signs, including one death. The virus titers shed by these birds with antibodies against NDV vary from 5.9 to 7.4 log_10_ EID_50_/mL. In the group of birds challenged with EG14 virus, four out of nine with positive antibodies against NDV displayed mild or moderate clinical signs, without any mortality. No clinical signs or mortality were recorded in the six birds with positive titers by the ELISA test at 1/100 dilution challenged with TZ12, but they shed virus at titers varying from 5.7 to 6.8 log_10_ EID_50_/mL. A positive correlation (Pearson r = −0.5189) was found when comparing the antibody titers measured by the ELISA test using the 1/100 dilution at 18 dpv and virus shedding at 4 dpc (*p < 0.001*, Figure S3). However, no antibody against NDV was detectable at 18 dpv by ELISA even at 1/100 dilution in five, two, and six birds vaccinated with adeno-F and clinically protected against the challenge with CA02, EG14, and TZ12 viruses, respectively. No significant difference was detected when compared to the ELISA titers at 1/100 dilution within the adeno-F-vaccinated groups. In summary, 94% (*n* = 16) of dead birds, 58% (*n* = 12) of sick birds, and 40% (*n* = 32) of healthy birds out of all challenged birds (*n* = 60) had no detectable antibodies against the F protein before the challenge. A significant difference was detected when compared to the ELISA titers from dead birds and healthy birds at 1/100 dilution in the group challenged with the EG14 strain (*p* = 0.021) and with the TZ12 strain (*p* = 0059). A significant difference was also noticed when compared to the ELISA titers from dead and healthy birds from all challenged groups (*p* < 0.0001). All surviving birds seroconverted with high HI and ELISA titers at 14 dpc.

## 4. Discussion

Live vaccines are commonly used in the poultry industry as they allow for the mass vaccination of a flock in the hatchery or the farm [39]. Live vaccines can induce an early immune response, which induces cellular, mucosal, and antibody responses [40]. However, the presence of an NDV-specific maternal antibody can reduce the immune response [41,42]. Our study agrees with previous studies showing that the LaSota vaccine at a high dose could provide complete protection against mortality and clinical signs [24,25]. The LaSota vaccine was also able to reduce virus shedding to low levels despite the amino acid differences between the vaccine and challenge viruses. The HI and ELISA tests detected a high systemic humoral response. Nevertheless, a small increase in the HI titers at 14 dpc shows that the virus replicated enough to stimulate an immune response in the birds. In summary, this vaccine remains an excellent tool to prevent clinical disease, but it is not able to inhibit virus replication in chickens and virus shedding to the environment. The major disadvantage of live vaccines is the potential for vaccine reactions, which may result in some clinical disease and increased condemnations at the processing plant [43].

Recombinant vector vaccines have been widely used in the poultry industry because they not only induce immunity against two pathogens, but they can also be used for differentiating infected from vaccinated animals (DIVA) strategy in the field [44]. The herpesvirus of turkeys and fowlpox recombinant vector vaccines for Newcastle disease virus were developed to express the F or HN NDV proteins [18,22,45]. Although the HN protein seems to be a major antigenic determinant of NDV [5], some studies that individually expressed the HN or F proteins showed that the fusion protein appears to provide better protection than HN to protect birds against an NDV challenge [15,17]. Here, we used human adenovirus serotype 5 (Ad5) that has a deletion of the delta E1 and E2 genes, impeding their replication and allowing the expression of the target protein for a limited time [46]. Transduction of just the F protein allows us to determine the importance of the protein for protective immunity, the dose effect, and the impact of the homology of this protein for the clinical protection against an NDV challenge. As has been demonstrated with other recombinant vector vaccines expressing just the F protein, an immune response to the F protein can provide clinical protection [18,47,48]. A clear dose effect was also observed with adeno-F, providing only partial protection with the low dose and much higher protection with the high dose, which had 10 times more adeno-F viral particles. This study also supports the value of homologous vaccination to just the F protein in providing better clinical protection and reduction in viral shedding. In previous studies, a whole virus (recombinant NDV or inactivated) vaccine homologous to the challenge virus could significantly reduce virus shedding by the oral route in comparison to heterologous vaccines [26]. The antigenic similarity of the HN protein also likely contributes to the better clinical protection and reduction in virus shedding [49]. In our study, the similarity of the fusion protein to the challenge strain seems to be important to improve the clinical protection and decrease virus shedding.

The present study also correlates the systemic humoral response before the challenge with clinical protection. The correlation between high neutralizing antibodies measured by the HI test and good clinical/virological protection is well accepted because high and more specific levels of antibodies to the NDV challenge strain are required to significantly decrease viral replication [24,50]. Antibodies against the F+HN protein transferred through passive immunity can also provide complete protection against an NDV challenge [51]. In our study, high HI antibody titers were detected in birds vaccinated with the live NDV vaccine which correlates with good protection. However, no antibody response to the adeno-F vaccine was detectable by the HI test, and still, partial protection was observed. This finding was expected as the HI test can measure the antibodies of the HN protein’s hemagglutination activity, which was not inserted in the adeno-F vaccine. Complete or partial protection against an NDV vaccine was already reported with the absence of HI antibodies after vaccination with recombinant vector vaccines expressing the F protein [18,47]. In this study, the majority of birds with detectable antibodies using an ELISA test against the F protein in the adeno-F groups, using less diluted sera, were clinically protected against the challenge. The antigen coated on the plates in the ELISA kit could cause differences in the correlation of the clinical protection and the immune response as the three viruses studied belong to different NDV genotypes. This finding can suggest the need for more sensitive tools to detect low titers of specific antibody responses to NDV, but a protective cellular immune response, which was not measured in this study, also likely played a role in protection [15,52]. Cell-mediated immunity has already been demonstrated to be activated by live NDV vaccines in chickens, and the replication-defective adenoviral vectors can elicit the cell-mediated response in mice and chickens [53,54,55,56].

## 5. Conclusions

This is the first study evaluating the antigenic similarity of only the fusion protein using NDV strains belonging to different genotypes. The adeno-F viral particle concentration and the similarity of the protein to the challenge virus affected the protection. For example, single transduction was able to provide partial protection to the challenged birds at an early challenge. Although virus shedding was reduced using the adeno-F vaccine when compared to the controls, the reduction was more pronounced when using a live vaccine. Our study supports the potential role of cell-mediated immunity in the clinical protection against a challenge with virulent NDV strains as the humoral response does not always correlate with NDV protection. This study also provides insight into NDV protection and will be helpful for the design and optimization of vectored vaccine platforms.

## Figures and Tables

**Table 1:** ELISA titers from birds vaccinated with adeno-F at high dose before and after challenge with the three different NDV isolates. Birds found dead or euthanized are highlighted in black and birds displaying any clinical signs are highlighted in dark gray. Positive sera at 18 days dpv and at 11 dpc by ELISA test using 1/500 or 1/100 dilutions are highlighted in green. ELISA was tested using 1/500 dilution at 11 dpc.

		18 dpv		11 dpc
Challenge Virus	Bird Tag	Titer (1/100)	Titer (1/500)	Titer (1/500)
CA02	**30**	1283	104	16,480
	**31**	779	0	15,545
	**32**	147	0	16,932
	**33**	3451	1128	15,453
	**34**	1155	197	NS
	**35**	809	394	16,274
	**36**	1291	389	16,214
	**37**	1674	482	NT
	**38**	2006	126	16,622
	**39**	2442	1008	16,377
	**40**	418	263	NS
	**41**	478	42	15,436
	**42**	711	201	NS
	**43**	3842	729	15,268
	**44**	1200	214	16,856
	**45**	1246	274	16,089
	**46**	613	0	NS
	**47**	297	0	14,779
	**48**	917	44	13,397
	**49**	290	69	17,900
	Mean	1252	283	15,974.8
EG14	**70**	147	11	15,464
	**71**	0	27	NS
	**72**	1125	0	13,903
	**73**	1396	125	13,827
	**74**	1373	687	14,110
	**75**	1109	21	13,713
	**76**	79	60	NS
	**77**	3195	860	14,716
	**78**	726	203	NS
	**79**	470	186	12,631
	**80**	282	219	NS
	**81**	34	93	NS
	**82**	102	246	NS
	**83**	681	76	NS
	**84**	1735	264	12,220
	**85**	147	7	16,078
	**86**	1321	49	14,879
	**87**	297	104	11,329
	**88**	1629	186	14,191
	**89**	2713	482	13,074
	Mean	928	258	13,857
TZ12	**110**	282	509	10,668
	**111**	237	60	NS
	**112**	64	88	11,652
	**113**	0	181	NS
	**114**	214	4	12,837
	**115**	2134	111	15,360
	**116**	478	31	14,132
	**117**	3533	729	15,154
	**118**	0	0	5549
	**119**	1795	236	14,955
	**120**	0	44	NS
	**121**	290	0	14,550
	**122**	8390	2613	14,096
	**123**	689	471	16,013
	**124**	0	307	NS
	**125**	0	0	NS
	**126**	493	0	12,704
	**127**	2585	323	15,083
	**128**	1915	159	14,787
	**129**	403	142	17,590
	Mean	1567	327	13,675

dpv: days post-vaccination, dpc: days post-challenge, NT: not tested, NS: non-surviving.

## Data Availability

The data presented in this study are openly available in https://doi.org/10.3390/vaccines9020182.

## Supplementary Materials

The following are available online at https://www.mdpi.com/2076-393X/9/2/182/s1, Figure S1: Experimental design of animal experiments. (A) Birds vaccinated with one of two doses (low or high) of adeno-F and non-vaccinated birds were challenged with CA02 virus at 3 weeks post-vaccination. The clinical signs and mortality were followed for 14 days. Samples were collected for performing the HI and RRT-PCR tests to evaluate the humoral response before and after challenge and virus shedding at 2 and 4 dpc. (B) Birds vaccinated with the live vaccine (LaSota) or with the high dose of adeno-F and non-vaccinated birds were challenged with three different viruses. The clinical signs and mortality were followed for 11 days. Samples were also collected to measure the humoral response before and after the challenge and virus shedding at 2, 4, and 7 dpc. Figure S2: MDCK cells labeled with polyclonal antibody against F NDV protein and FITC anti-rabbit after 48 h after infection/transduction. (A) Negative control; (B) cells infected with LaSota; (C) cells transduced with adeno-F (200X). Figure S3: The correlation between virus titers in sera and virus shedding from birds vaccinated with adeno-F at 1/100 dilution was observed. All titers measured before the challenge with one of the three viruses are plotted, as well as the virus shedding at 4 dpc by the oral route, except from bird 122 which has a titer over 8000.
