# Supplementary material for: Protection against Different Genotypes of Newcastle Disease Viruses (NDV) Afforded by an Adenovirus-Vectored Fusion Protein and Live NDV Vaccines in Chickens
Source: Vaccines (Basel). 2021 Feb 21;9(2):182. doi: 10.3390/vaccines9020182 (PMC7924635; doi:10.3390/vaccines9020182)

Journal: Vaccines- Special Issue on Vaccine Research against Significant Viral Diseases of Poultry

Article: Protection against different genotypes of Newcastle disease viruses (NDV) afforded by an adenovirus-vectored fusion protein and live NDV vaccines in chickens

Authors: Helena L. Ferreira, Patti J. Miller, David L. Suarez

Supplemental Figure 1. Experimental design of animal experiments. A) Birds vaccinated with one of two doses (low or high) of adeno-F and non-vaccinated birds were challenged with the CA02 virus at 3 weeks post vaccination. The clinical signs and mortality were followed for 14 days. Samples were collected for performing the HI and RRT-PCR tests to evaluate the humoral response before and after challenge and the virus shedding at 2 and 4 dpc. B) Birds vaccinated with the live vaccine (LaSota) or with the high dose of adeno-F and non-vaccinated birds were challenged with three different viruses. The clinical signs and mortality were followed for 11 days. Samples were also collected to measure the humoral response before and after the challenge and virus shedding at 2, 4, and 7 dpc.

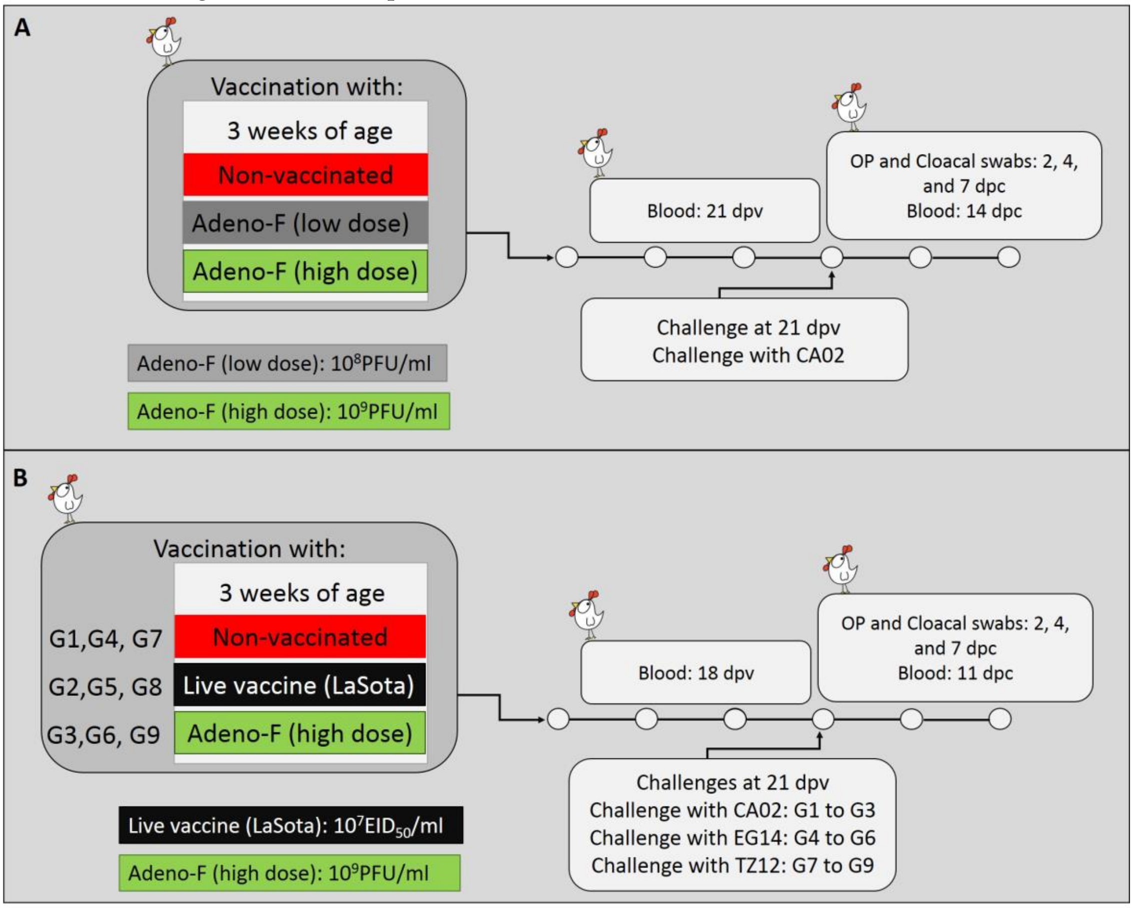

Supplement: Supplementary file 1 [file vaccines-09-00182-s001.zip › Supplemental Figure 1.pdf]
