# Supplementary material for: Protection against Different Genotypes of Newcastle Disease Viruses (NDV) Afforded by an Adenovirus-Vectored Fusion Protein and Live NDV Vaccines in Chickens
Source: Vaccines (Basel). 2021 Feb 21;9(2):182. doi: 10.3390/vaccines9020182 (PMC7924635; doi:10.3390/vaccines9020182)

Journal: Vaccines- Special Issue on Vaccine Research against Significant Viral Diseases of Poultry

Article: Protection against different genotypes of Newcastle disease viruses (NDV) afforded by an adenovirus-vectored fusion protein and live NDV vaccines in chickens

Authors: Helena L. Ferreira, Patti J. Miller, David L. Suarez

Supplemental Figure 2. MDCK cells labelled with polyclonal antibody against F NDV protein and FITC anti-rabbit after 48 hours after infection/transduction. A) Negative control; B) cells infected with LaSota; C) cells transduced with adeno-F (200X).

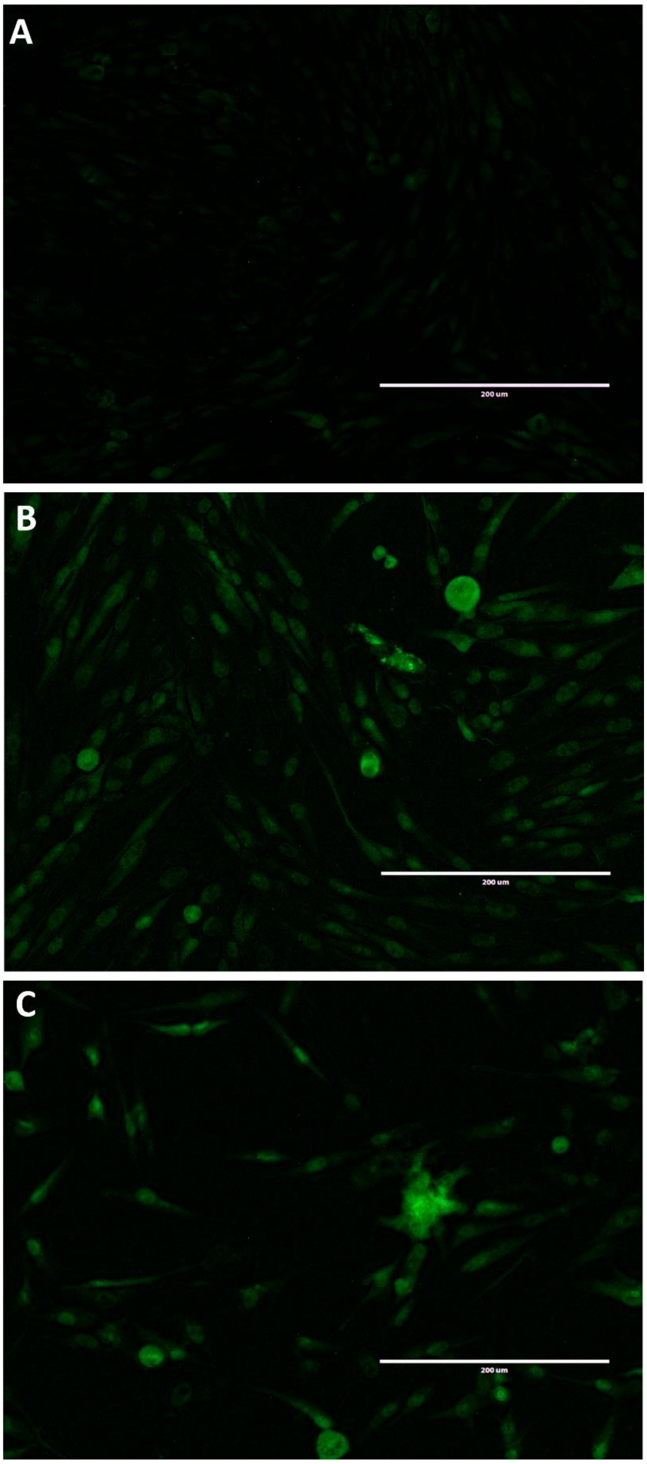

Supplement: Supplementary file 1 [file vaccines-09-00182-s001.zip › Supplemental Figure 2.pdf]
