# Supplementary material for: Protection against Different Genotypes of Newcastle Disease Viruses (NDV) Afforded by an Adenovirus-Vectored Fusion Protein and Live NDV Vaccines in Chickens
Source: Vaccines (Basel). 2021 Feb 21;9(2):182. doi: 10.3390/vaccines9020182 (PMC7924635; doi:10.3390/vaccines9020182)

**Journal:** Vaccines- Special Issue on Vaccine Research against Significant Viral Diseases of Poultry

**Article:** Protection against different genotypes of Newcastle disease viruses (NDV) afforded by an adenovirus-vectored fusion protein and live NDV vaccines in chickens

**Authors:** Helena L. Ferreira, Patti J. Miller, David L. Suarez

**Supplemental Figure 3.** The correlation between virus titers in sera and virus shedding from birds vaccinated with adeno-F at 1/100 dilution was observed. All titers measured before the challenge with one of the three viruses were plotted and the virus shedding at 4 dpc by oral route, except from bird 122 which has a titer over 8,000.

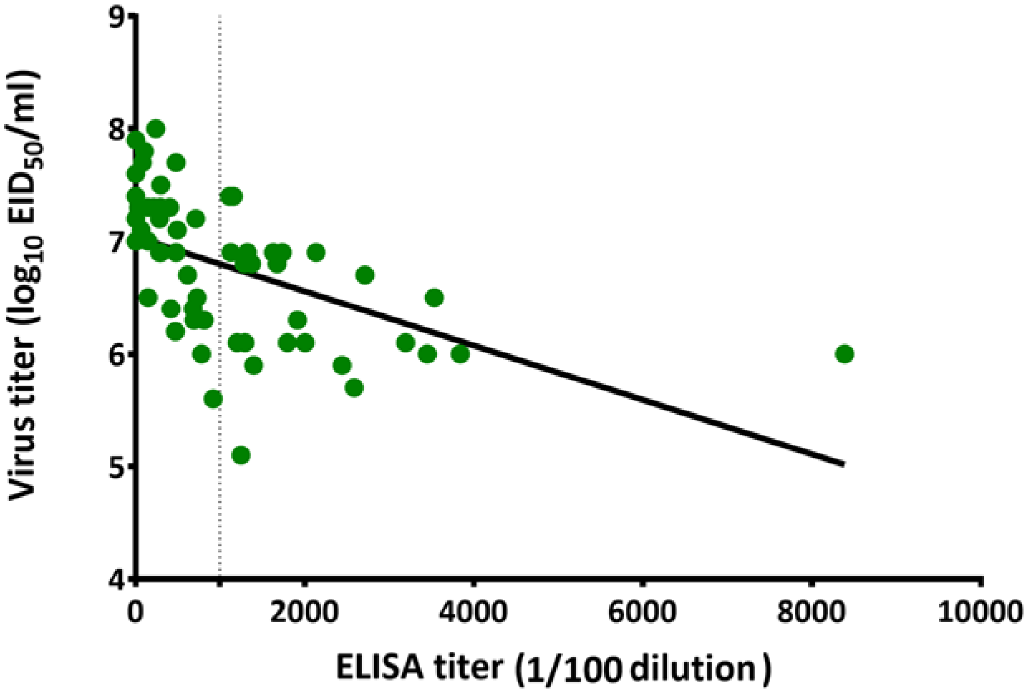

Supplement: Supplementary file 1 [file vaccines-09-00182-s001.zip › Supplemental Figure 3.pdf]
